# Decade Growing in Plain Sight: Detection of an Invasive Melanoma Within a Long-Standing Pigmented Lesion

**DOI:** 10.7759/cureus.117734

**Published:** 2026-10-10

**Authors:** Marlena Freeman, Gregory Gates, Iris Ahronowitz

**Affiliations:** 1 Dermatology, David Geffen School of Medicine, University of California at Los Angeles (UCLA), Los Angeles, USA; 2 Dermatopathology, David Geffen School of Medicine, University of California at Los Angeles (UCLA), Los Angeles, USA

**Keywords:** delayed melanoma diagnosis, melanoma, melanoma arising in lentigo, missed melanoma, occult melanoma

## Abstract

Cutaneous melanoma remains the leading cause of skin cancer mortality, and accurate diagnosis depends on representative biopsy of suspicious pigmented lesions. Large, clinically atypical lesions on the lower extremity are particularly prone to sampling error, and chronic stasis change and background photodamage can further confound histopathologic interpretation. We report the case of a woman in her 60s with a remote history of lentigo maligna of the left cheek who presented for routine skin examination and was found to have a nearly 7 cm diameter variegated brown plaque on the right calf, with two previous partial biopsies returning a diagnosis of benign lentigo. A broader re-biopsy revealed invasive melanoma arising in a background of florid melanoma in situ with focal regression and a Breslow thickness of at least 1.0 mm. Wide local excision demonstrated residual melanoma in situ with clear margins, and sentinel lymph node biopsy identified rare Melanoma Antigen Recognized by T cells 1 (MART-1) positive cells of indeterminate significance in a single right deep inguinal node. This case illustrates the limitations of partial biopsy in broad pigmented lesions, the prognostic ambiguity of focal regression, and the challenging clinical interpretation of rare immunohistochemistry-positive cells in sentinel lymph nodes. It reinforces the importance of clinicopathologic correlation and a low threshold for broader re-biopsy when focal sampling of a clinically atypical lesion repeatedly returns benign histologically.

## Introduction

Cutaneous melanoma is a malignancy arising from the melanocytes of the skin and is driven primarily by exposure to ultraviolet radiation from natural sunlight and indoor tanning [1]. Primary lesions are commonly darkly pigmented but may also be amelanotic, representing 2-8% of melanoma cases [2]. The mainstay of treatment for primary melanoma is wide local excision with margins determined by tumor thickness; additional treatment is guided by the disease stage as defined by histopathologic examination and sentinel lymph node biopsy when indicated [1].

The incidence of cutaneous melanoma in the United States is approximately 30 cases per 100,000 population [2]. Accurate histopathologic assessment of suspicious pigmented lesions depends on: an appropriate biopsy technique, adequate clinical information provided to the pathologist, and interpretation by a pathologist experienced in melanocytic neoplasms. Representative tissue sampling is essential, and partial biopsy techniques can substantially underestimate or fail to identify melanoma in large or heterogeneous lesions [3,4]. Clinically atypical pigmented lesions on the lower extremity of older patients present a particular diagnostic challenge, as background photodamage, stasis change, and pigment incontinence can mimic or mask melanocytic neoplasia [5,6].

## Case presentation

A woman in her 60s presented to the dermatology clinic for a routine total body skin examination. Her dermatologic history was notable for a lentigo maligna of the left cheek diagnosed over 30 years earlier and treated with Mohs micrographic surgery, with no evidence of recurrence on subsequent surveillance. Her past medical history was significant for hypertension, hyperlipidemia, and prior smoking history.

Examination of the right lower extremity revealed a 6.8 cm irregular, variegated brown plaque with indistinct borders located on the posterior aspect of the right calf (Figures 1, 2).

**Figure 1:**
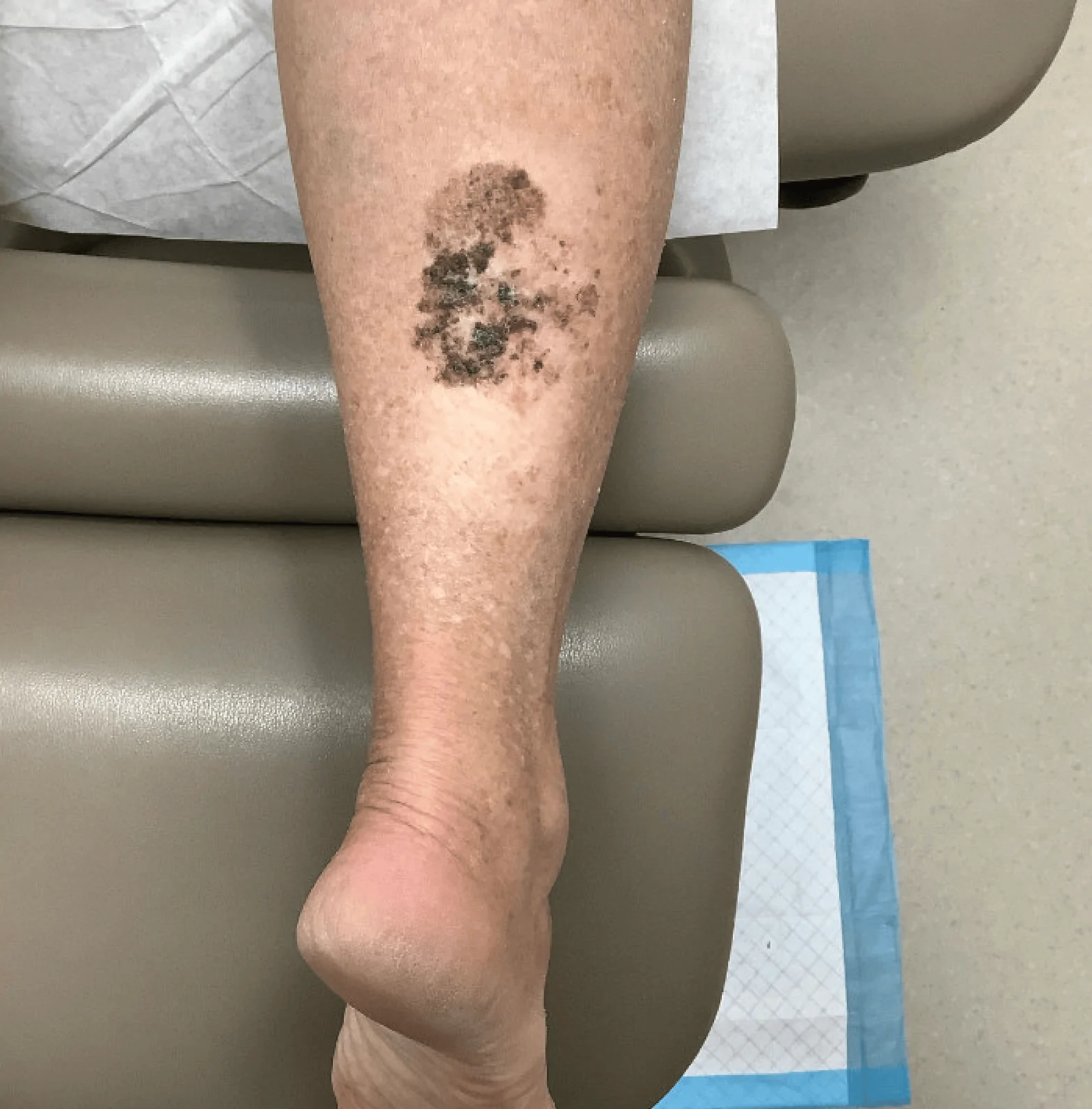
Broad pigmented plaque on the lower extremity, exhibiting variegated color, areas of hypopigmentation, and irregular borders

**Figure 2:**
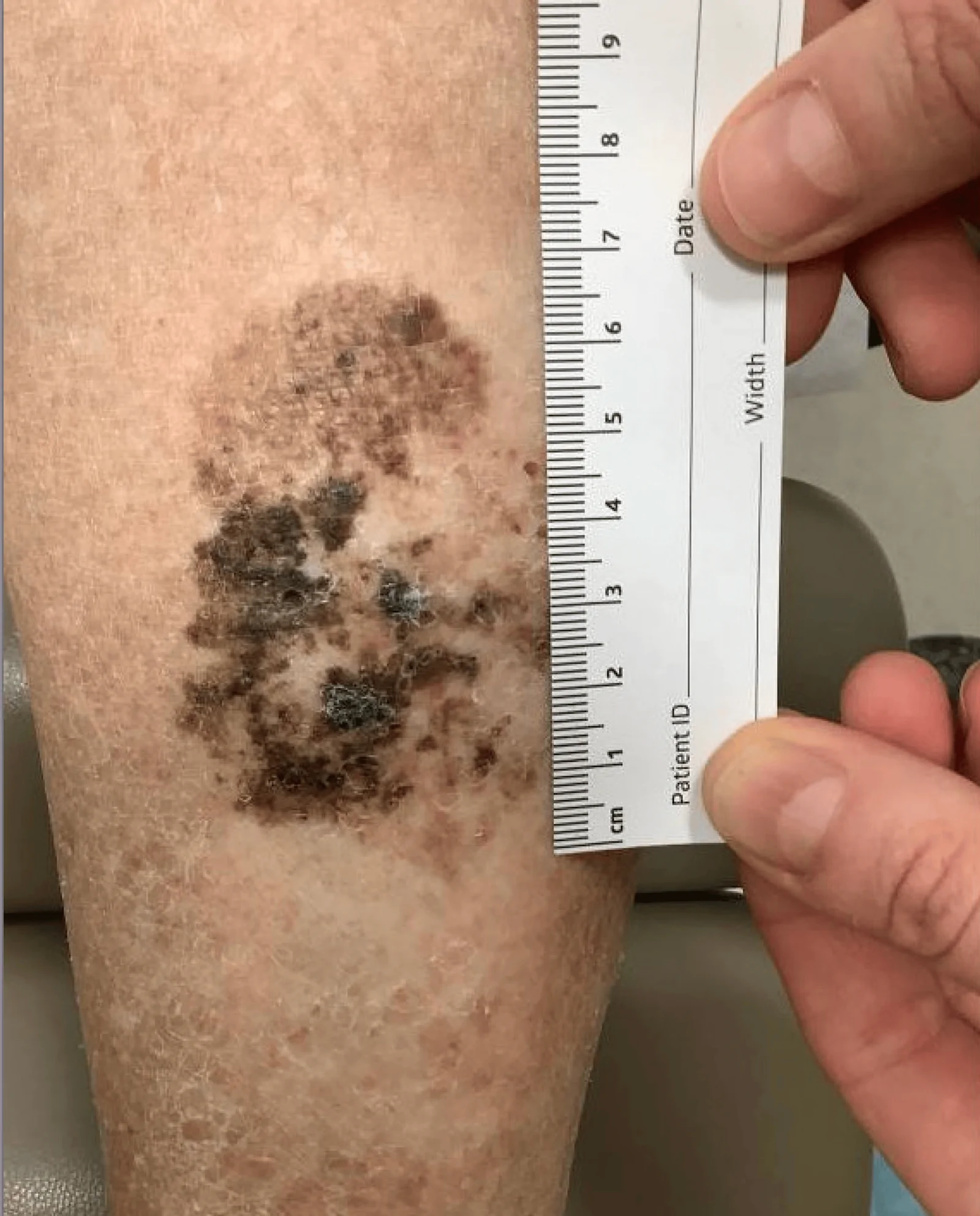
Closer view of broad pigmented plaque on the lower extremity, exhibiting variegated color, areas of hypopigmentation, and irregular borders

The lesion had been clinically followed for several years and had been biopsied twice. A 1 cm presumed seborrheic keratosis was first noted at this site, 17 years prior. In the preceding 14 years, the lesion was described as a 3 cm asymmetric brown patch with indistinct borders, and a scouting partial shave biopsy at that time returned a diagnosis of lentigo without melanocytic atypia. Ten years prior, the lesion appeared subtly darker; a repeat partial scouting shave biopsy again demonstrated benign lentigo.

Given the persistent clinical atypia and progressive enlargement of the lesion, a broader re-biopsy was recommended. The patient was referred to a dermatologic surgeon for a larger, more representative re-biopsy. Histopathologic examination of this broader specimen revealed melanoma arising in a background of florid melanoma in situ with focal regression (Figure 3).

**Figure 3:**
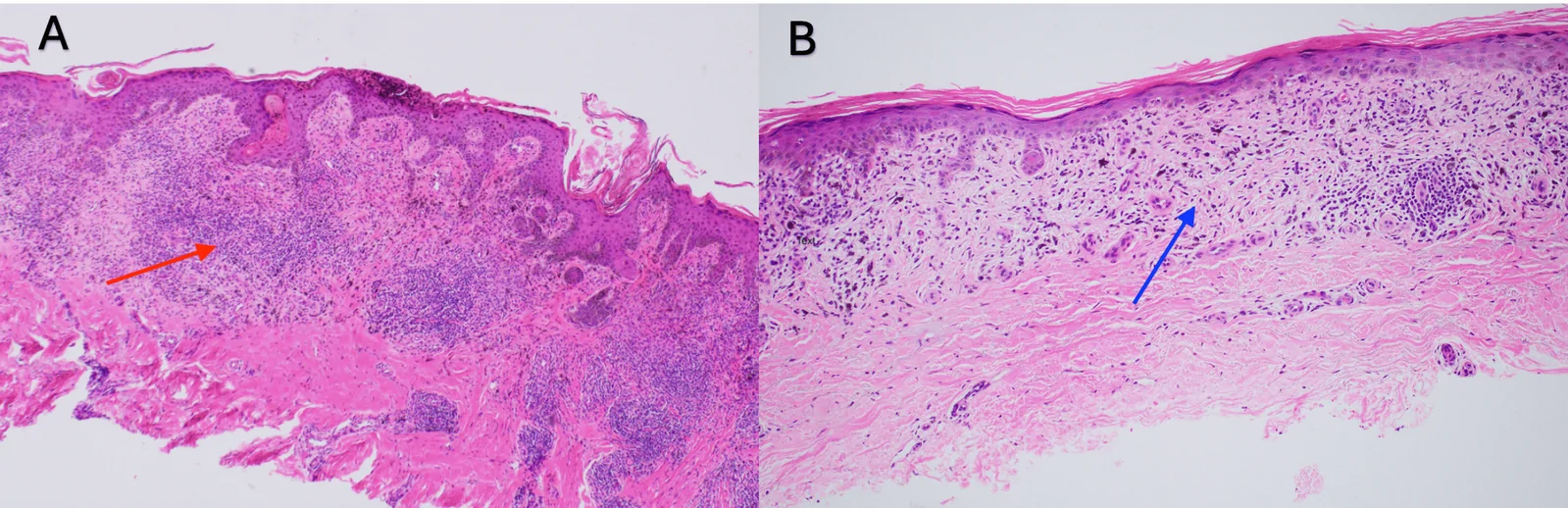
Excisional biopsy histopathology Hematoxylin and eosin (H&E) stain demonstrating invasive melanoma (red arrow) with areas of regression (blue arrow): A) 4x magnification; B) 10x magnification.

Breslow thickness was reported as at least 1.0 mm, with lesional cells extending to the biopsy edges. Background features included focal dermal fibrosis and stasis change.

One month following the diagnosis, the patient underwent wide local excision of the right calf lesion with 1.5 cm clinical margins and selective right deep inguinal sentinel lymph node biopsy. Final pathology of the wide excision specimen demonstrated residual melanoma in situ with surgical margins that appeared clear on examined sections. Sentinel lymph node biopsy was performed using preoperative lymphoscintigraphy. Histopathologic examination of the sentinel lymph node (measuring 2.3 x 2.0 x 1.0 cm) identified approximately 14 cells that stained positively with Melanoma Antigen Recognized by T-cells 1 (MART-1) centrally within the node, not apparent on hematoxylin and eosin (H&E) stain (Figure 4).

**Figure 4:**
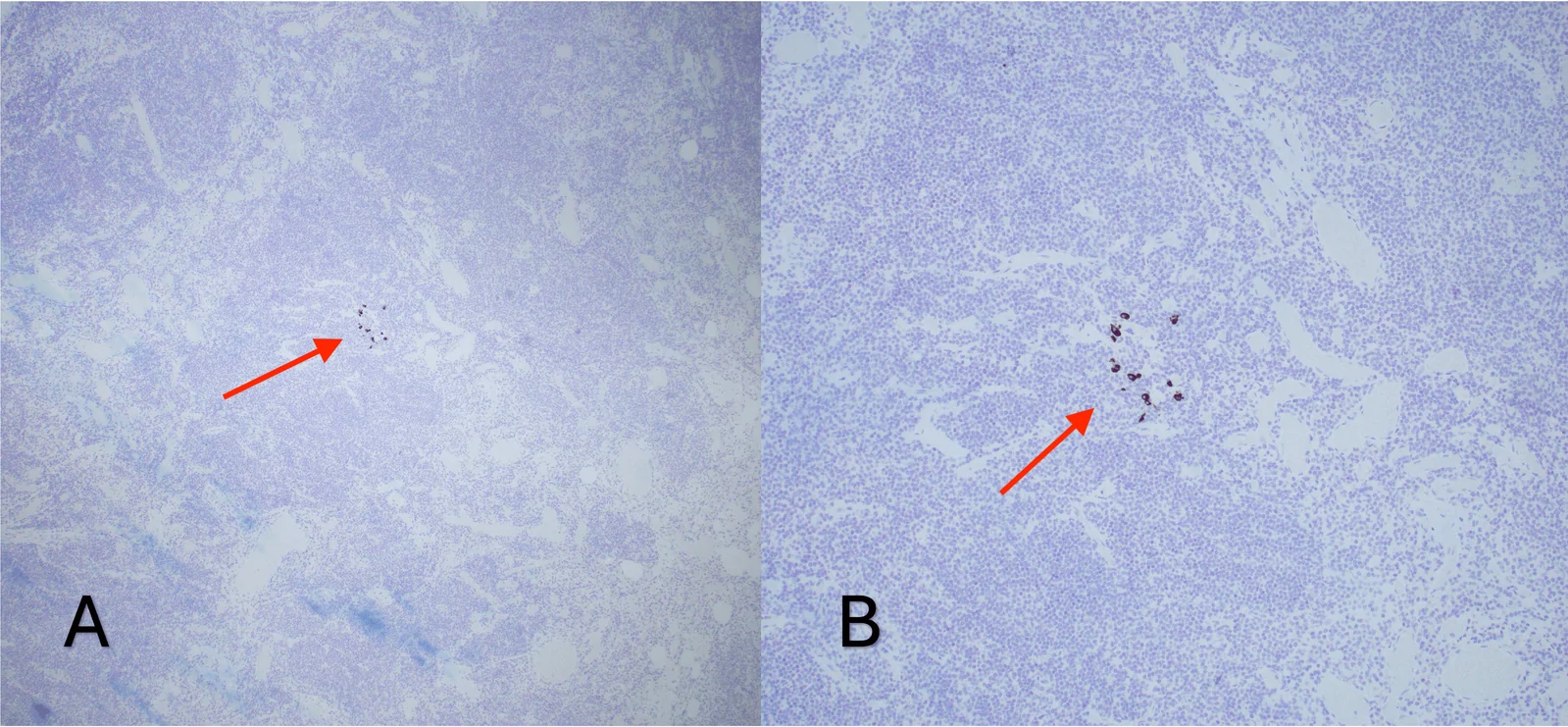
Sentinel lymph node biopsy histopathology MART-1 (Melanoma Antigen Recognized by T-cells 1) staining reveals a focus of melanocytes not apparent on hematoxylin and eosin (H&E) stain, denoted by red arrows:  A) 4x magnification; B) 10x magnification.

Subsequent attempts with Sex-determining Region Y-box transcription factor 10 (SOX10) and tyrosinase immunohistochemical stains failed to highlight the same focus. The findings were reported as suspicious for, but not diagnostic of, metastatic melanoma.

A preoperative ultrasound of the right lower extremity demonstrated a cluster of small popliteal lymph nodes measuring up to 6 mm in short axis with preserved fatty hila, consistent with reactive change and recommended for surveillance imaging (Figure 5).

**Figure 5:**
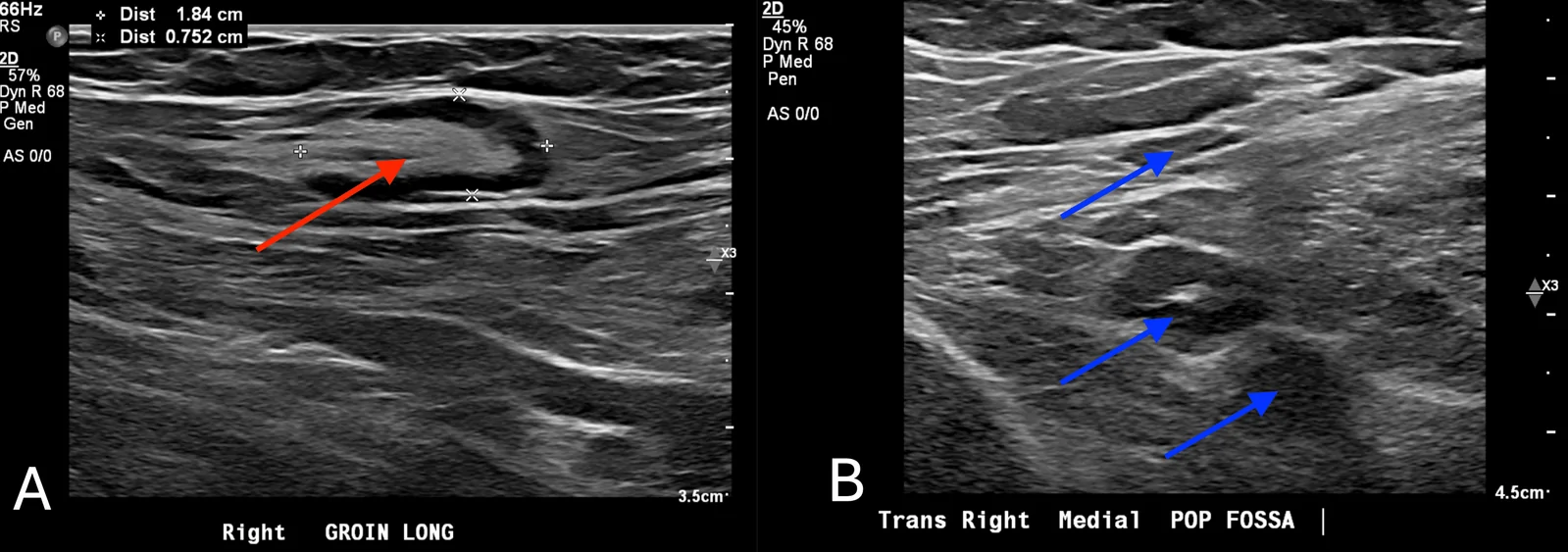
Preoperative ultrasound images of lymph nodes A) Imaging of the right groin shows a normal looking lymph node with a fatty hilum and thin cortex, measuring 7 mm in short axis presumably corresponding to the sentinel node (red arrow); B) Imaging of the popliteal fossa demonstrates a small cluster of indeterminate nodes up to 6 mm in short axis but with slightly thickened cortices consistent with reactive lymph nodes (blue arrows).

The patient subsequently underwent a split-thickness skin graft of the right lower leg following the wide local excision. At the time of this report, she remains under close dermatologic, surgical oncology, and plastic surgery follow-up with planned interval clinical examinations including skin checks every three months.

## Discussion

This case illustrates several diagnostic challenges in the workup of a clinically atypical pigmented lesion of the lower extremity: the limitations of partial biopsy in large lesions, the implications of focal histologic regression, and the interpretation of rare immunohistochemistry-positive cells in sentinel lymph nodes.

The diagnostic delay in this case reflects a well-described limitation of partial biopsy techniques in clinically heterogeneous or evolving pigmented lesions. In a series of 1,783 melanoma patients, Karimipour et al. demonstrated that when substantial clinical lesion remained after incisional biopsy, mean Breslow thickness increased from 0.66 mm on initial biopsy to 1.07 mm on excision, with upstaging occurring in 21% of patients and 10% becoming newly eligible for sentinel lymph node biopsy [3]. A prospective Australian series found that, compared with excisional biopsy, punch biopsy carried a 16.6-fold increased odds of histopathologic misdiagnosis [4].

To preserve lesional architecture and allow complete histopathologic assessment, the American Academy of Dermatology recommends excisional or complete biopsy as the preferred approach for suspicious pigmented lesions, with partial or incisional biopsy reserved for large lesions or challenging anatomic sites and explicitly targeted at the clinically thickest or most atypical area [7]. Superficial shave biopsies are generally discouraged when melanoma is suspected, with the notable exception of suspected lentigo maligna, for which a broad shave extending into the deep papillary or superficial reticular dermis may be appropriate [7].

In our patient, two prior partial scouting shave biopsies over a 14-year interval had returned diagnoses of benign lentigo before a broader, more representative re-biopsy revealed invasive melanoma. This sequence highlights the value of clinicopathologic persistence: when a lesion remains clinically suspicious despite reassuring histology, broader resampling is warranted, particularly in lesions that evolve over time.

Interpretation of melanocytic lesions on the lower extremity is further complicated by background histopathologic changes that can develop in skin with chronic photodamage or venous insufficiency, regardless of patient age. Chronic venous stasis is associated with dermal melanophages, pigment incontinence, and reactive epidermal changes, all of which can mimic or obscure melanocytic neoplasia [5,8]. Our patient's specimen was reported with background stasis change and focal dermal fibrosis, both of which may have contributed to the difficulty of earlier histopathologic interpretation.

Focal regression was identified in the diagnostic biopsy. Regression in melanoma is characterized histologically by dermal lymphocytic infiltrate, fibrosis, melanophages, and epidermal attenuation, and is reported in approximately 10% to 35% of cutaneous melanomas [9]. Its prognostic significance remains controversial, with some series suggesting a favorable association with sentinel lymph node status and others finding no independent effect on nodal metastasis or melanoma-specific survival [10,11]. A separate clinical concern is that regression may cause underestimation of true Breslow thickness by thinning previously deeper tumors [9]. In this case, the reported Breslow thickness of at least 1.0 mm with focal regression and lesional cells extending to biopsy edges suggests that the true tumor depth may have been greater than measured, which is relevant both for staging and surveillance planning.

Finally, the sentinel lymph node biopsy in this case identified approximately 14 cells that stained positive for MART-1 centrally within the node, which were not detected on subsequent contiguous sections or with SOX10 or tyrosinase staining. Nodal nevi, typically located in the capsule or trabeculae, are present in approximately 22% of sentinel lymph node specimens from melanoma patients and do not carry adverse prognostic significance [12]. In contrast, isolated tumor cells within the parenchyma have been shown to confer modestly reduced melanoma-specific survival compared with negative sentinel nodes, with five-year survival of 89% versus 94% in one large series [13]. A more recent series of 821 sentinel lymph node biopsies identified 57 cases (6.9%) with rare immunohistochemistry-positive cells of undetermined clinical significance, which demonstrated disease-specific and recurrence-free survival comparable to negative sentinel nodes over a mean follow-up of 59 months [14]. The absence of staining on SOX10 and tyrosinase in our patient, along with the inability to demonstrate the MART-1 positive cells on deeper levels, supports an interpretation of indeterminate significance rather than definitive metastasis. The Second Multicenter Selective Lymphadenectomy Trial (MSLT-II) trial established that even for confirmed sentinel lymph node micrometastasis, observation with nodal ultrasound is non-inferior to immediate completion lymphadenectomy for melanoma-specific survival [15], a finding that informs the current preference for close surveillance over additional, potentially morbid surgery, in cases of low-burden or indeterminate nodal disease.

## Conclusions

This case illustrates the diagnostic challenges posed by large, clinically atypical pigmented lesions on the lower extremity. Repeated partial biopsies over more than a decade returned diagnoses of benign lentigo before broader resampling revealed invasive melanoma arising in a background of florid melanoma in situ with focal regression. Clinicians should maintain a high index of suspicion for melanoma in long-standing, clinically atypical pigmented lesions, particularly in patients with a prior history of melanoma, and should pursue broader or larger sampling for re-biopsy when clinical concern persists despite reassuring partial biopsy histology. Interpretation of sentinel lymph node findings in such cases requires careful clinicopathologic correlation, and lifelong surveillance remains essential given the elevated risk of recurrence or additional primary disease.
